# Anti-hyperglycemic contours of Madhugrit are robustly translated in the *Caenorhabditis elegans* model of lipid accumulation by regulating oxidative stress and inflammatory response

**DOI:** 10.3389/fendo.2022.1064532

**Published:** 2022-12-05

**Authors:** Acharya Balkrishna, Vivek Gohel, Nishit Pathak, Meenu Tomer, Malini Rawat, Rishabh Dev, Anurag Varshney

**Affiliations:** ^1^ Drug Discovery and Development Division, Patanjali Research Institute, Governed by Patanjali Research Foundation Trust, Haridwar, Uttarakhand, India; ^2^ Department of Allied and Applied Sciences, University of Patanjali, Haridwar, Uttarakhand, India; ^3^ Patanjali Yog Peeth (UK) Trust, Glasgow, United Kingdom; ^4^ Special Centre for Systems Medicine, Jawaharlal Nehru University, New Delhi, India

**Keywords:** Madhugrit, ayurveda, diabetes, inflammation, wound healing, lipid accumulation, oxidative stress

## Abstract

**Background:**

The prevalence of diabetes has considerably increased in recent years. In the long run, use of dual therapy of anti-diabetic agents becomes mandatory to attain euglycemia. Also, the incidences of diabetes-related co-morbidities have warranted the search for new therapeutic approaches for the management of the disease. Traditional herbo-mineral, anti-diabetic agents like Madhugrit are often prescribed to mitigate diabetes and related complications. The present study aimed to thoroughly characterize the pharmacological applications of Madhugrit.

**Methods:**

Phytometabolite characterization of Madhugrit was performed by ultra-high performance liquid chromatography. Evaluation of cell viability, α-amylase inhibition, glucose uptake, inflammation, and wound healing was performed by *in vitro* model systems using AR42J, L6, THP1, HaCaT cells, and reporter cell lines namely NF-κB, TNF-α, and IL-1β. The formation of advanced glycation end products was determined by cell-free assay. In addition, the therapeutic potential of Madhugrit was also analyzed in the *in vivo Caenorhabditis elegans* model system. Parameters like brood size, % curling, glucose and triglyceride accumulation, lipid deposition, ROS generation, and lipid peroxidation were determined under hyperglycemic conditions induced by the addition of supraphysiological glucose levels.

**Results:**

Madhugrit treatment significantly reduced the α-amylase release, enhanced glucose uptake, decreased AGEs formation, reduced differentiation of monocyte to macrophage, lowered the pro-inflammatory cytokine release, and enhanced wound healing in the *in vitro* hyperglycemic (glucose; 25 mM) conditions. In *C. elegans* stimulated with 100 mM glucose, Madhugrit (30 µg/ml) treatment normalized brood size, reduced curling behavior, decreased accumulation of glucose, triglycerides, and lowered oxidative stress.

**Conclusions:**

Madhugrit showed multimodal approaches in combating hyperglycemia and related complications due to the presence of anti-diabetic, anti-inflammatory, anti-oxidant, wound healing, and lipid-lowering phytoconstituents in its arsenal. The study warrants the translational use of Madhugrit as an effective medicine for diabetes and associated co-morbidities.

## Introduction

Diabetes is a fast-growing chronic metabolic disorder of global concern. Currently, about 463 million people have diabetes which has been estimated to increase to 700 million by the year 2045 (1, 2). The sub-optimal glycemic control in diabetes has been linked to persistent systemic inflammation, immune cell dysfunction, and poor wound healing (3). An effective therapeutic approach to manage diabetes must include control of postprandial hyperglycemia, utilization of excess glucose, minimization of protein glycation, oxidative stress, and inflammation (4–6). The current therapeutic agents can only address one part of the pathologic manifestations, leaving a considerable portion of diabetic complications unchecked. Since multiple problems are associated with the pathophysiology and treatment of diabetes, the use of monotherapy is not sufficient (6, 7). The long-term use of pharmacological agents for the treatment of diabetes can also incur adverse effects and treatment resistance, mandating dose titration and inclusion of additional drugs (7–9).

The α-amylase, a calcium metalloenzyme, causes postprandial hyperglycemia by increasing the digestion of ingested carbohydrates (10, 11). A sustained increase in glucose levels leads to the development of oxidative stress which is reported to be responsible for inflammation, impaired glucose utilization in peripheral tissues, and delayed wound healing (4, 7, 12, 13). During diabetes, an alteration occurs in the gut microbiota which leads to the release of microbial products like lipopolysaccharide (LPS). These events are believed to cause low-grade inflammation, mediated by the induction of inflammatory cytokines by gut immune cells (14). Hence, an effective therapeutic agent for diabetes must be able to subside hyperglycemia, oxidative stress, and inflammation to control diabetes and related symptoms.

The phytopharmaceutical product Madhugrit is routinely prescribed to diabetic individuals for the maintenance of euglycemia. Madhugrit is composed of Chandraprabha Vati (classical Ayurvedic medicine) (15), supplemented with Giloy (*Tinospora cordifolia*), Indrayana (*Citrullus colocynthis*), Karela (*Momordica charantia*), Chirayata (*Swertia chirata*), Shatavar (*Asparagus racemosus*), Ashwagandha (*Withania somnifera*) and Shuddh Shilajit (*Asphaltum punjabianum*) (**Supplementary Tables 1, 2**). Due to the presence of these anti-diabetic (16–18), anti-oxidant (19–21), and anti-inflammatory (22) components, Madhugrit might be able to maintain glucose levels efficiently and manage other diabetes related complications.

At present, the pre-clinical evaluation of anti-diabetic agents is carried out by chemical, surgical, and genetic manipulations on rodents (23). A disadvantage of such models is that they are time-consuming, expensive, and require comprehensive regulatory approvals (24). The nematode *Caenorhabditis elegans* is being increasingly used as an acceptable *in vivo* model of human diseases. This model of the multiorgan eukaryotic organism has several benefits like small size (~1 mm length of an adult), substantial progeny (~300 by parthenogenesis), and a short lifespan (~21 days). Furthermore, research using *C. elegans* does not need extensive regulatory approvals. *C. elegans* has been substantially used in studies of glucose-induced toxicity (24, 25). Consequently, *C. elegans* model could also be used to screen anti-diabetic test articles (26).

The present study investigated the effect of Madhugrit on alleviating diabetic complications induced by hyperglycemic conditions. Extensive phytochemical profiling of Madhugrit was carried out by HPLC analysis and subsequently, its pharmacological activity was explored. The therapeutic potential of Madhugrit was evaluated by *in vitro* analysis of parameters such as cell viability, modulations in α-amylase release, glucose uptake, inflammation, wound healing, and formation of advanced glycation end products (AGEs). These *in vitro* experiments were followed by *in vivo* analysis in *C. elegans* by measuring brood size, % curling, glucose levels, triglyceride accumulation, lipid deposition, ROS generation and lipid peroxidation. The biguanide anti-hyperglycemic drug, Metformin, was used as the method control (27–30), in parallel.

## Materials and methods

### Reagents

Madhugrit (batch #1MDT-210081) was sourced from Divya Pharmacy, India. The standards for HPLC analysis namely gallic acid, magnoflorine, piperine, rutin, ellagic acid, coumarin, cinnamic acid, and palmatine were obtained from Sigma-Aldrich, USA; protocatechuic acid, corilagin from Natural remedies, India, and methyl gallate from TCI chemicals, India. Reagents namely RPMI 1640 (no glucose), DMEM (low glucose), DPBS, antibiotic-antimycotic solution, starch, Malondialdehyde (MDA), Trichloroacetic acid (TCA), LPS, and 2′,7′-Dichlorofluorescin diacetate (H_2_DCFDA) were procured from Sigma-Aldrich, USA. Heat-inactivated FBS, TPVG, Bovine serum albumin (BSA), Nile red, and Alamar blue were obtained from HiMedia, India. Horse serum was bought from Gibco, USA. Chemicals, 2-thiobarbituric acid (TBA), D-glucose, Metformin, and dexamethasone were obtained from TCI chemicals, India. Phorbol 12-myristate 13-acetate (PMA) was purchased from Alfa Aesar, UK. Mitomycin C was obtained from Roche, Germany. Pierce BCA protein assay kit and sodium azide were obtained from Thermo Fisher Scientific, USA. Human TNF-α and IL-6 ELISA kits were obtained from BD Biosciences, USA. QUANTI-Blue reagent was purchased from InvivoGen, USA.

### Phytochemical analysis of Madhugrit on UHPLC platform

Madhugrit powder(500 mg) was diluted with 10 ml water: methanol (50:50), sonicated for 30 min, centrifuged at 10,000×g for 5 min, and filtered by a 0.45 µm nylon filter. This solution was further used for the metabolite analysis. Prominence-XR UHPLC system (Shimadzu, Japan) equipped with a Quaternary pump (Nexera XR LC-20AD XR), DAD detector (SPD-M20 A), auto-sampler (Nexera XR SIL-20 AC XR), degassing unit (DGU-20A 5R) and column oven (CTO-10 AS VP) were used for analysis. Separation was achieved using a Shodex C18-4E (5µm, 4.6 X 250 mm) column subjected to binary gradient elution. The two solvents used for the analysis were: (A) water containing 0.1% orthophosphoric acid (pH 2.5) and (B) 0.1% orthophosphoric acid in a mixture of acetonitrile and water (88:12). Gradient programming of the solvent system: 5% B for 0-5 min, 5-10% B from 5-20 min, 10% B from 20-30 min, 10-20% B from 30-40 min, 20-35% B from 40-50 min, 35-60% B from 50-65 min, 60-85% B from 65-70 min, 85-5% B from 70-71 min and 5% B from 71-75 min with a flow rate of 1 ml/min. A 10 µl of standard and test solution was injected and the column temperature was maintained at 30°C. Wavelength of 270 nm was used for gallic acid, methyl gallate, protocatechuic acid, magnoflorine, corilagin, coumarin, cinnamic acid, piperine, and palmatine; 250 nm for ellagic acid and 350 nm for rutin. The obtained chromatograms of standards and sample were overlayed and the phytometabolites were quantified respectively at different retention times (RT).

### Cell culture maintenance

The rat pancreatic cell line AR42J; rat myoblast cells L6; human monocytic cells THP-1 were procured from the ATCC licensed repository, National Centre for Cell Science, India. The human keratinocyte cells HaCaT were purchased from Krishgen Biosystems, India. The reporter cell lines namely HEK-Blue TNF-α, HEK-Blue IL-1β, and THP1-Blue NF-κB were obtained from *In vivo*Gen, USA. AR42J, L6, and HaCaT were propagated in normal glucose (NG, 5.5 mM) DMEM supplemented with 10% FBS and 1% antibiotic-antimycotic solution. THP-1 cells were cultured in normal NG (5.5 mM) RPMI 1640 supplemented with 10% FBS and 1% antibiotic-antimycotic solution. The reporter cell lines were cultured as per the manufacturer’s instructions. The cultured cells were maintained at 37°C and 5% CO_2_ in a humidified incubator and used within 5 passages after revival.

### Cell viability

The effect of Madhugrit on the viability of AR42J, L6, THP-1 monocytes, THP1 macrophages, and HaCaT, were evaluated by Alamar blue dye (15 µg/ml). Madhugrit (10, 30, 100 µg/ml) was added during differentiation of AR42J into amylase-secreting exocrine cells by 100 nM dexamethasone and incubated for 72 hr (31–33). The L6 cells were differentiated with 2% Horse serum containing media for 72 hr post which the cells were incubated with Madhugrit (10-100 µg/ml) for 24 hr. THP1 monocytes and macrophages were incubated with Madhugrit (10-100 µg/ml) for 48 hr. HaCaT cells were also treated for 48 hr with Madhugrit (10-100 µg/ml). Following incubation with Alamar blue dye, the plates were read at Ex. 560/Em.590 nm by Envision multimode plate reader (PerkinElmer, USA). All treatments were performed with NG (5.5 mM) culture media. Data were presented as mean ± SEM (n=3).

### Assessment of α-amylase inhibition in AR42J cells

AR42J cells were plated at a density of 1×10^6^ cells per well in a 6-well plate. Post 72 hr treatment, as described earlier, cells were washed with DPBS and induced with 1% (w/v) starch for 1 hr after which the supernatant was collected and α-amylase (U/L) levels were analyzed by Architect ci8200 biochemical analyzer (Abbott Diagnostics, USA), at an external laboratory. Undifferentiated cells (UDC) were used as normal control. Data were presented as mean ± SEM (n=3).

### Assessment of glucose uptake in L6 cells

L6 cells were seeded at a density of 2×10^5^ cells per well in 12-well plate. After differentiation, cells were treated with Madhugrit (10, 30, and 100 µg/ml) or Metformin (2 mM) in serum-free NG (5.5 mM) culture media. After 24 hr the cells were washed with DPBS and stimulated with 100 µM 2-NBDG dye for 1 hr in DPBS, thereafter cells were washed twice with DPBS and lysed by 2 phased freeze-thaw process. The cell lysate was collected, centrifuged at 12,000×g for 10 min and 100 µl of the supernatant was pipetted in 96-well black plate and read at Ex.475/Em.529 nm by Envision multimode plate reader (PerkinElmer, USA). Data were presented as mean ± SEM (n=3).

### Advanced glycation end products (AGEs) assessment

The evaluation of the anti-glycation activity of Madhugrit was done as per Starowicz et al. (34) with slight modifications. Briefly, glucose (90 mg/ml) and BSA (10 mg/ml) were separately dissolved in DPBS. Then, Madhugrit (0-100 µg/ml) or Metformin (2 mM) were mixed with a BSA-glucose solution. The use of 0.01% sodium azide was done to prevent microbe development. After 1 week of incubation at 37°C, the fluorescence of AGEs was measured at Ex.350/Em.420 nm and the inhibition of AGEs formation was evaluated. Data were presented as mean ± SEM (n=3).

### Assessment of THP-1 monocyte (suspension cells) to macrophage (adherent cells) differentiation

THP-1 monocytes (5×10^5^ cells) were pre-treated with Madhugrit (10, 30, and 100 µg/ml) or Metformin (2 mM) in T-25 flasks for 24 hr. Afterward, the cells were centrifuged, washed with DPBS, and plated at a density of 3×10^4^ cells/well in a 96-well plate. The cells were again treated with Madhugrit or Metformin in presence of 20 ng/ml PMA for 24 hr. All treatments were performed with NG (5.5 mM) culture media. Post incubation the cells were washed and Alamar blue assay was performed. Data were presented as mean ± SEM (n=3).

### Assessment of LPS-induced TNF-α and IL-6 cytokine release in THP-1 macrophages

THP-1 monocytes were plated at a density of 1×10^5^ cells per well. The cells were differentiated to macrophages and pre-treated with Madhugrit (10, 30, and 100 µg/ml) or Metformin (2 mM) for 24 hr. Following incubation, the cells were washed and stimulated with 100 ng/ml LPS in presence of Madhugrit (10, 30, and 100 µg/ml) or Metformin (2 mM) for 24 hr. Untreated control (UC) group was taken as normal control. All treatments were performed with NG (5.5 mM) culture media. The cell supernatant was then collected and assessed for TNF-α and IL-6 release by sandwich ELISA as per the manufacturer’s instructions. Data were presented as mean ± SEM (n=4).

### Assessment of LPS-induced NF-κB, TNF-α, and IL-1β activity under high glucose conditions

THP-1 macrophages were pre-treated with Madhugrit (10, 30, and 100 µg/ml) or Metformin (2 mM) for 24 hr. Later on, the cells were stimulated for 24 hr by LPS (500 ng/ml) and divided into 5 groups: (1) UC NG (5.5 mM), (2) LPS NG (5.5 mM), (3) UC HG (25 mM), (4) LPS HG (25 mM) and (5) LPS HG (25 mM) with Madhugrit (10, 30, 100 µg/ml) or Metformin (2 mM). After 24 hr the cell supernatant was collected and incubated with the NF-κB, TNF-α, and IL-1β reporter cells. Subsequently, based on the secreted embryonic alkaline phosphatase (SEAP) levels released by the reporter cells the activity of NF-κB, TNF-α and IL-1β was evaluated by QUANTI-Blue reagent as per the manufacturer’s instructions. The plates were read at an optical density of 630 nm by Envision multimode plate reader. Data were presented as mean ± SEM (n=3).

### Scratch wound healing assay

HaCaT cells (2×10^5^/well) were cultured in 12 well plates and pre-treated with Madhugrit (10, 30, 100 µg/ml) or Metformin (2 mM) for 24 hr. Later on, the cells were washed with DPBS and induced with mitomycin C (5 µg/ml) to inhibit proliferation. The cells were washed and wounded by a 10 µl plastic pipette tip and further rinsed with DPBS to remove cell debris. The cells were treated and divided into 5 groups: (1) UC NG (5.5 mM), (2) UC HG (25 mM), and (3) HG (25 mM) with Madhugrit (10, 30, 100 µg/ml) or Metformin (2 mM). Images were acquired at 0 hr and 24 hr. Analysis of % wound closure and rate of migration (µm/hr) were done by Fiji, ImageJ (NIH, USA) (13, 35). Data were presented as mean ± SEM (n=3).

### Maintenance and treatment of *Caenorhabditis elegans*

**Figure 1:**
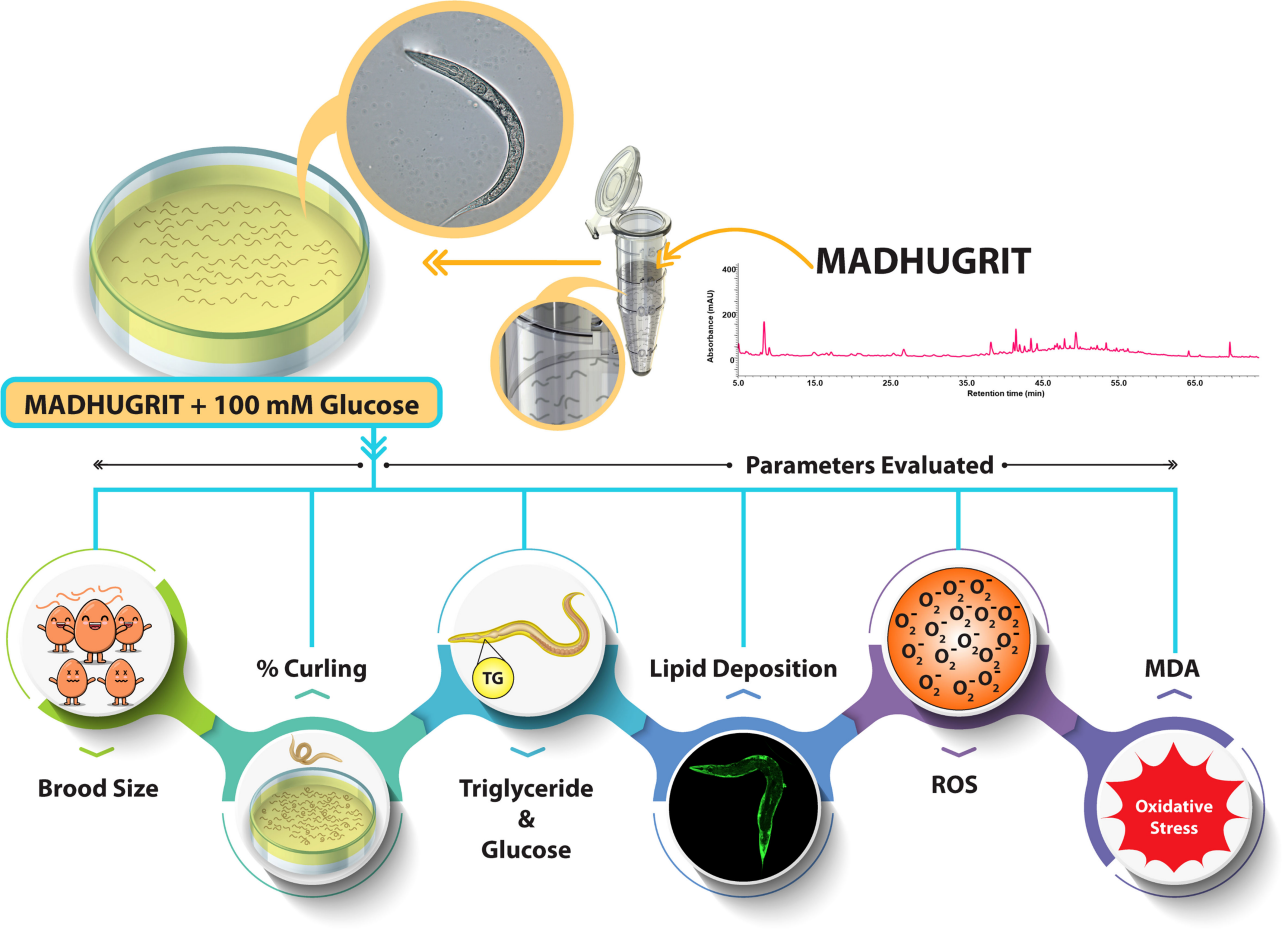
Schematic of *C. elegans* treatment and parameters evaluated.

The N2 (wild type) *C. elegans* strain was procured from the Caenorhabditis Genetics Center (CGC) and maintained in NGM (nematode growth medium) seeded with *E. coli* OP50 at 20°C. A synchronization technique was used to separate the eggs from the worms by treatment with an alkaline hypochlorite solution (25, 36). The hatched eggs released the L1 larvae, used for exposure to various treatments. The L1 nematodes were exposed to different concentrations of Madhugrit (3, 10, and 30 μg/ml) or Metformin (2 mM) for 24 hr. After incubation, the nematodes were transferred to an NGM plate seeded with *E. coli* OP50 with or without high glucose (HG, 100 mM) supplementation along with Madhugrit (3, 10, and 30 μg/ml) or Metformin (2 mM). Treatment scheme and parameters analyzed on *C. elegans* experimental model are depicted in **Figure 1**.

### Brood size assessment

The nematodes were treated as described above and were maintained on NGM/*E. coli* OP50 plates until they reached larval stage L4. To evaluate progeny size, one nematode from each treated group was transferred to a new NGM plate seeded with *E. coli* OP50 with or without 100 mM glucose supplementation along with Madhugrit (3, 10, and 30 μg/ml) or Metformin (2 mM). The total number of progenies were counted by ZEISS Stemi 305 stereo microscope (Carl Zeiss, Germany). Data were presented as mean ± SEM (n=5).

### Analysis of aberrant behavior (curling) in C. elegans

After pre-treatment, 30 worms of the L4 stage were transferred to new NGM plates seeded with *E. coli* OP50 with or without 100 mM glucose supplementation along with Madhugrit (3, 10, and 30 μg/ml) or Metformin (2 mM). The worms that displayed a curling behavior (37) were counted by ZEISS Stemi 305 stereo microscope (Carl Zeiss, Germany). Data were presented as mean ± SEM (n=5).

### Assessment of glucose and triglyceride accumulation in *C. elegans*

The pre-treated L4 stage nematodes were transferred to a new NGM plate seeded with *E. coli* OP50 with or without 100 mM glucose supplementation along with Madhugrit (3, 10, and 30 μg/ml) or Metformin (2 mM). On Day 5, worms were washed off from the plates and centrifuged at 600×g for 1 min. Worms were washed rigorously and placed in lysis buffer (Tris 100 mM, NaCl 150 mM, EGTA 1 mM, EDTA 1 mM, Triton X-100 1%, and sodium deoxycholate 0.5%) supplemented with protease inhibitor cocktail (Thermo Fisher Scientific, USA) and passed through three cycles of freeze-thaw and centrifuged at 14000×g for 10 min. The supernatant was collected and analyzed for glucose and triglyceride levels by Erba 200 biochemical analyzer (Erba Mannheim, Germany). Protein concentration was determined using the Pierce BCA protein assay kit. The obtained values were further normalized with protein concentration. Data were presented as mean ± SEM (n=3).

### Microscopy of lipid deposition in *C. elegans* by Nile red stain

After treatment, worms were washed off from the plates and centrifuged at 600×g for 1 min. The worms were washed thrice and fixed with 40% isopropanol for 3 min, centrifuged at 600×g for 1 min, and stained with Nile red as per the protocol of Escorcia et al. (38). The images were acquired using a FITC filter on an Olympus BX43 microscope equipped with a Mantra imaging platform (PerkinElmer, USA) and further processed on Inform 2.2 software suite (PerkinElmer, USA).

### Evaluation of ROS levels in *C. elegans*

ROS levels in *C. elegans* were detected by the H_2_DCFDA dye as mentioned by Zhou et al. (39) with slight modifications. Briefly, worms were lysed, centrifuged and the supernatant was incubated with H_2_DCFDA (200 µM) at 37°C for 1 hr. The plates were read at Ex. 495/Em.523 nm by Envision multimode plate reader and the fluorescence values were normalized with protein concentration (40). Data were presented as mean ± SEM (n=3).

### Assessment of lipid peroxidation in *C. elegans*

The MDA content from the lysate of treated nematodes was measured by the TBA-TCA method (41). The optical density was read at 532 nm by Envision multimode plate reader and the amount of MDA present in the samples was determined from the standard curve. The data was further normalized with protein concentration. Data were presented as mean ± SEM (n=3).

### Data analysis

Statistical analysis was performed using one-way ANOVA with Dunnett’s multiple comparisons *post-hoc* test. Data were analyzed using GraphPad Prism 7 (GraphPad Software, USA). Results were considered to be statistically significant at a probability level of p < 0.05.

## Results

### Phytometabolite analysis of Madhugrit

**Figure 2:**
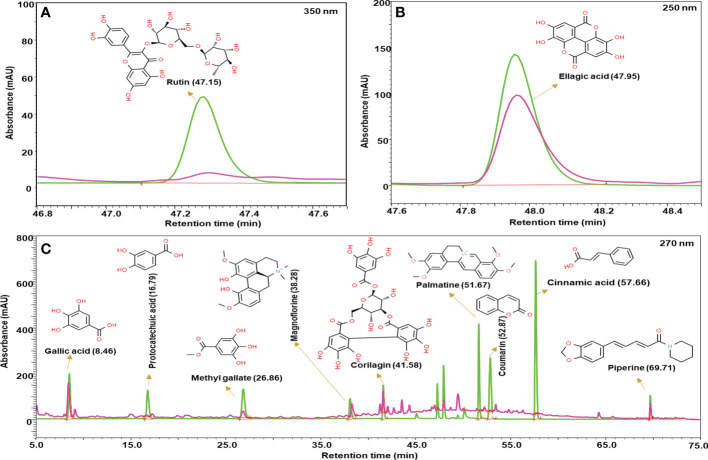
Phytometabolite analysis of Madhugrit by HPLC. Overlap chromatogram of standards (green line) and Madhugrit (pink line). Rutin was quantified at 350 nm **(A)**, Ellagic acid at 250 nm **(B)**, and Gallic acid, Protocatechuic acid, Methyl gallate, Magnoflorine, Corilagin, Palmatine, Coumarin, Cinnamic acid, and Piperine at 270 nm wavelength **(C)**.

The quantitative analysis of phytometabolites using standard marker compounds (**Figure 2**) confirmed the presence of rutin, ellagic acid, gallic acid, protocatechuic acid, methyl gallate, magnoflorine, corilagin, palmatine, coumarin, cinnamic acid and piperine in Madhugrit. The quantitative analysis of phytochemicals using reference standards is mentioned in **Table 1**.

**Table 1:** Quantitative analysis of phytometabolites in Madhugrit.

Sr. No.	Compound name	Retention time (min)	Amount (in µg/mg)
**1**	Gallic acid	8.46	1.158
**2**	Magnoflorine	38.28	0.950
**3**	Corilagin	41.58	0.770
**4**	Piperine	69.71	0.449
**5**	Methyl gallate	26.86	0.305
**6**	Rutin	47.15	0.278
**7**	Ellagic acid	47.95	0.198
**8**	Protocatechuic acid	16.79	0.081
**9**	Coumarin	52.87	0.026
**10**	Cinnamic acid	57.66	0.021
**11**	Palmatine	51.67	0.014

HPLC quantified major metabolites present in as deciphered from the chromatogram shown in **Figure 2**.

### Cell viability analysis of Madhugrit

**Figure 3:**
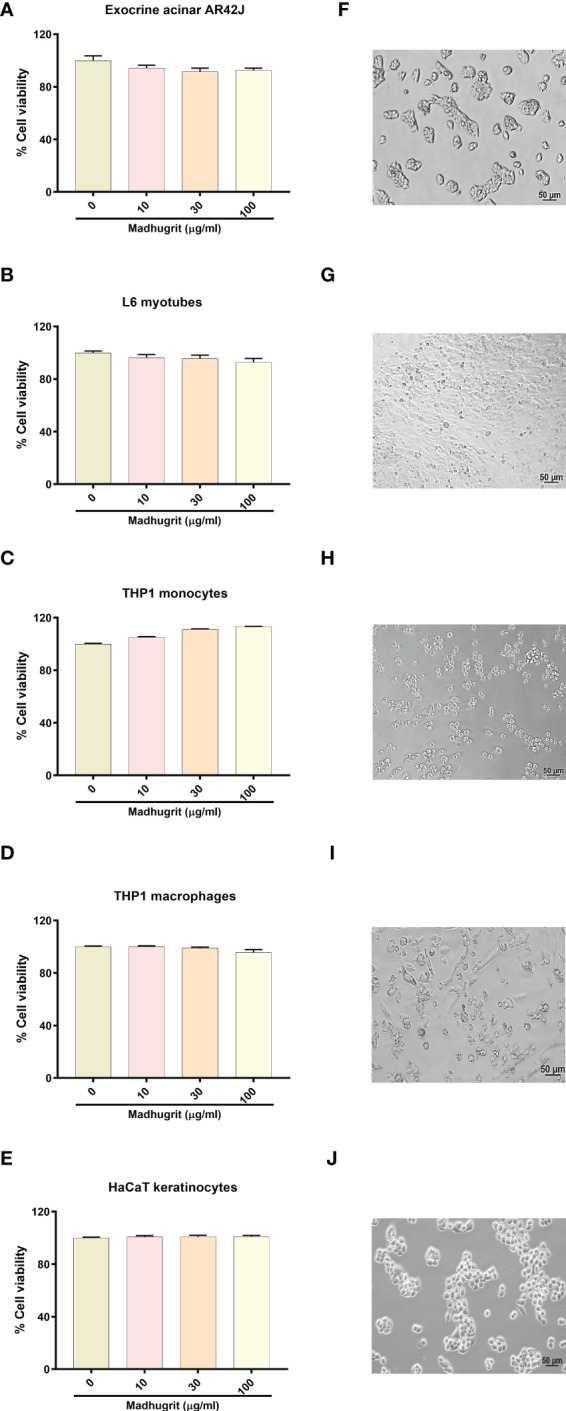
Cell viability analysis of Madhugrit. Cell viability of Madhugrit (10, 30, and 100 µg/ml) was performed on Exocrine acinar AR42J cells **(A)**, L6 myotubes **(B)**, THP1 monocytes **(C)**, THP1 macrophages **(D)** and HaCaT keratinocytes **(E)** by Alamar blue assay. Corresponding representative images of the cell lines used for the analysis **(F-J)**.

The effect of Madhugrit on the viability of cells was assessed on rat pancreatic exocrine like AR42J cells (**Figures 3A, F**), rat L6 myotubes (**Figures 3B, G**), human monocytic THP1 cells (**Figures 3C, H**), human THP1 macrophages (**Figures 3D, I**) and human HaCaT epidermal keratinocytes (**Figures 3E, J**). The cell lines were subjected to various concentrations of Madhugrit (10, 30, and 100 µg/ml) and no significant decrease in cell viability was observed even at 100 µg/ml concentration in all the cell lines. These preliminary results indicated that Madhugrit is non-toxic at all physiologically relevant concentrations and has no effect on the metabolic functionality of cells.

### Madhugrit reduced α-amylase secretion from pancreatic acinar-like cells and increased glucose uptake in skeletal myotubes

**Figure 4:**
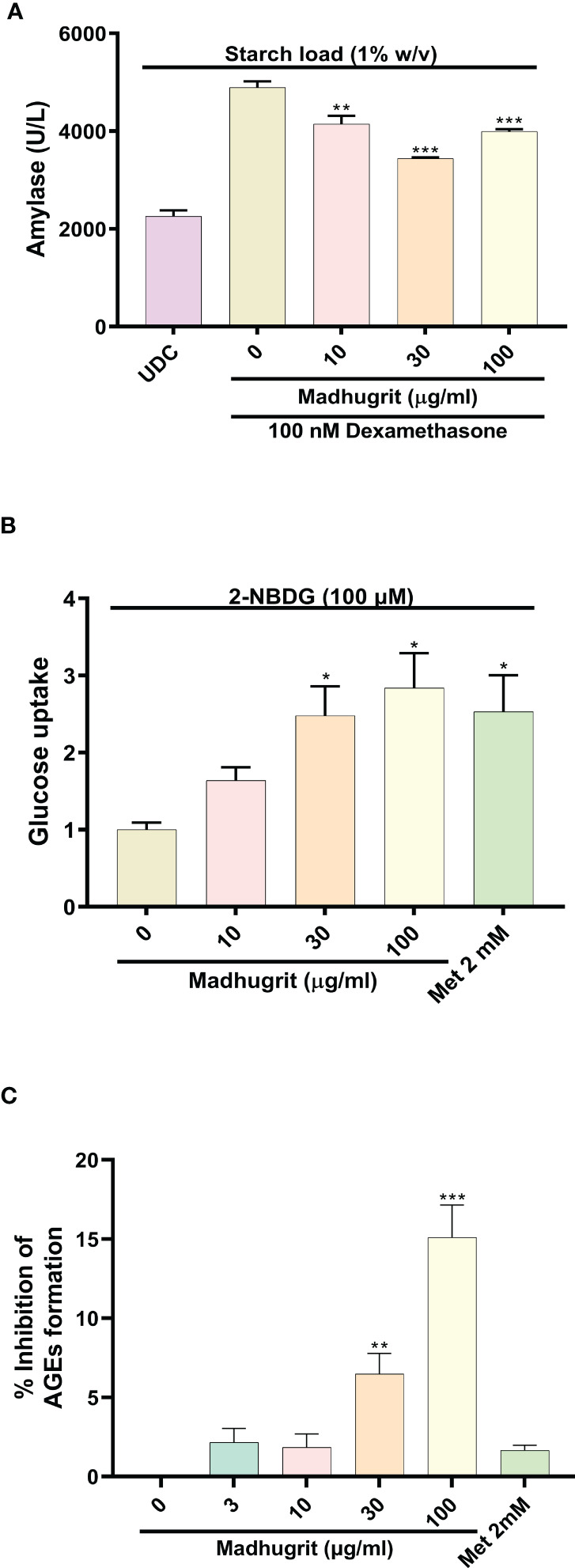
Analysis of amylase release, glucose uptake and inhibition of AGEs in presence of Madhugrit. **(A)** The release of amylase in dexamethasone differentiated AR42J cells was performed after induction with starch (1% w/v) in presence of Madhugrit. Undifferentiated cells (UDC) were taken as a normal control. **(B)** The uptake of glucose post Madhugrit treatment was evaluated in L6 myotubes by the fluorescent glucose analog 2-NBDG. **(C)** Evaluation of inhibitory potential of Madhugrit against AGEs formation. The statistical significance of the observed differences in the means compared to the Madhugrit (0 µg/ml) group was analyzed through one-way ANOVA followed by Dunnett’s multiple comparison test and represented as *, ** or *** depending on whether the calculated p value was <0.05, <0.01 or <0.001.

The AR42J cells was differentiated into an exocrine acinar-like pancreatic cell line (31) as the undifferentiated cells secrete less α-amylase in response to starch (**Figure 4A**). The bioactivity of Madhugrit in presence of starch load which mimics the physiological postprandial condition was determined on the differentiated cells. It was observed that Madhugrit (10, 30, and 100 µg/ml) treated cells released significantly (p <0.01) less α-amylase compared to control (**Figure 4A**) in response to 1% (w/v) starch. Next, to confirm the hypoglycemic activity of Madhugrit we evaluated its effect on glucose uptake levels in L6 cells (42). When compared with the control group, Madhugrit treatment with 10, 30, and 100 µg/ml significantly (p <0.05) promoted the uptake of glucose by 1.64, 2.48, and 2.84-fold respectively, in a concentration-dependent manner (**Figure 4B**). The results were at par with the positive control drug Metformin (2 mM) which also significantly (p <0.05) enhanced the glucose uptake levels by 2.53-fold compared to normal control.

### Madhugrit inhibited the *in vitro* AGEs formation

Advanced glycation end products (AGEs) are formed in high amounts during diabetes which get accumulated and develop oxidative stress (43). It was observed that Madhugrit (3-100 µg/ml) inhibited the *in vitro* formation of AGEs in a concentration-dependent manner (**Figure 4C**). An inhibition of more than 15% was observed in the Madhugrit (100 µg/ml) treated group. Hence, it was established that Madhugrit possesses a property to inhibit AGEs formation.

### Madhugrit decreased inflammation by inhibition of monocyte to macrophage differentiation and release of TNF-α and IL-6 cytokines

**Figure 5:**
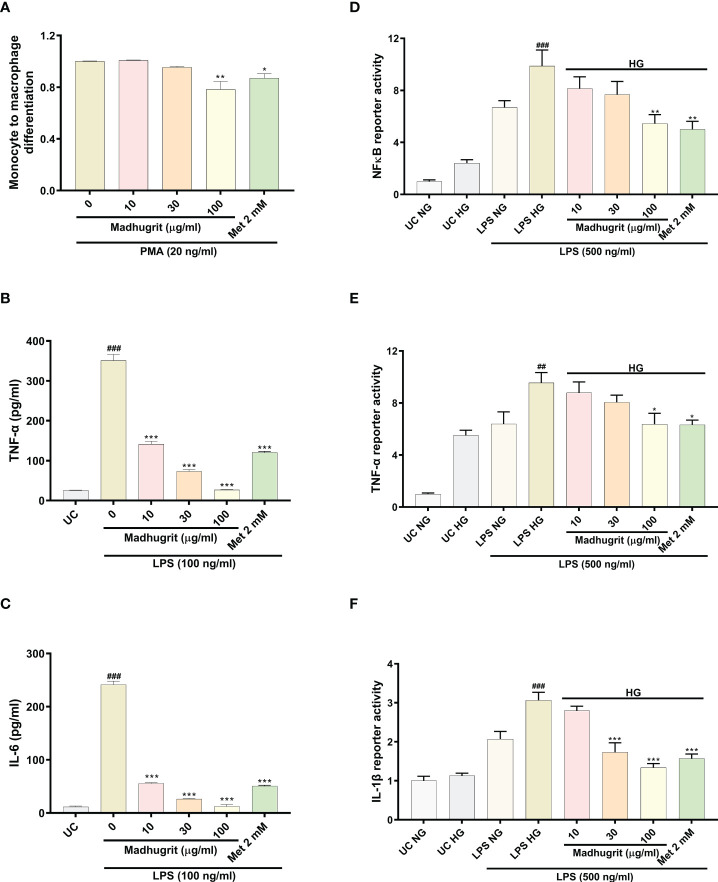
Madhugrit reduced monocyte to macrophage differentiation, release and activity of pro-inflammatory cytokines and NF-κB response. **(A)** Madhugrit inhibited the THP1monocyte to macrophage conversion in presence of PMA (20 ng/ml) represented as fold change. **(B, C)** The levels of released TNF-α and IL-6 (pg/ml) in presence of Madhugrit (0-100 µg/ml) or Metformin (2 mM) after LPS (100 ng/ml) induction. **(D-F)** Activity of NF-κB, TNF-α and IL-1β under normal glucose (NG) and high glucose (HG) conditions in presence of Madhugrit (0-100 µg/ml) or Metformin (2 mM) after LPS (500 ng/ml) induction. The statistical significance of the observed differences in the means compared to the **(A)** Madhugrit (0 µg/ml) was analyzed through one-way ANOVA followed by Dunnett’s multiple comparison test and represented as * (p<0.05) or ** (p<0.01). Statistical significance between Untreated control (UC) and Madhugrit (0 µg/ml) group **(B, C)** is represented as ### (p<0.001) and between UC HG and LPS HG group **(D-F)** as ## (p<0.01) or ### (p<0.001). Comparison of the treatments with Madhugrit (0 µg/ml) or LPS HG group was represented as *, ** or *** depending on whether the calculated p value was <0.05, <0.01 or <0.001.

The differentiation of monocytes to macrophages is a critical event that activates various pro-inflammatory signaling networks. The anti-inflammatory properties of Madhugrit were confirmed by the use of the *in vitro* model of THP1 monocyte to macrophage differentiation by PMA. It was observed that compared to control, Madhugrit at the concentration of 100 µg/ml significantly (p <0.01) decreased the conversion of monocytes to macrophages in presence of PMA (**Figure 5A**) as depicted by Alamar blue assay. Similarly, a significant (p <0.05) decrease was also observed in Metformin (2 mM) treated cells which was also previously reported by Vasamsetti et al. (28). Furthermore, the levels of LPS induced pro-inflammatory cytokines namely TNF-α and IL-6 were also evaluated post-Madhugrit treatment. An increase in the levels of LPS is observed due to an imbalance of gut microbiota during diabetes. LPS upon binding to toll-like receptor 4, found on immune cells activates a series of pro-inflammatory cascades, generally accompanied by an increase in levels of TNF-α and IL-6 (14, 44, 45). Madhugrit (10, 30, and 100 µg/ml) treated THP1 macrophages inhibited the release of LPS (100 ng/ml) induced TNF-α and IL-6 release, in a concentration-dependent manner (**Figures 5B, C**). The inhibitory effect of Madhugrit at 100 µg/ml concentration was more pronounced than Metformin (2 mM) in the case of TNF-α (**Figure 5B**) and IL-6 release (**Figure 5C**). Taken together, Madhugrit has anti-inflammatory properties, evident by its potential to inhibit macrophage differentiation and release of pro-inflammatory cytokines which is helpful to control the low-grade inflammation during diabetes.

### Madhugrit inhibited the activity of NF-κB, TNF-α, and IL-1β in THP-1 macrophages under hyperglycemic conditions

Macrophages under hyperglycemic state constantly express a pro-inflammatory phenotype which is more prominent in presence of exogenous stimulants (endotoxins) (3). In high glucose (25 mM) conditions, it was observed that THP1 macrophages without any LPS (500 ng/ml) challenge, compared to normal glucose (5.5 mM) conditions, showed an increase in their pro-inflammatory phenotype which became more pronounced upon LPS stimulation (**Figures 5D–F**). It was observed that Madhugrit (10, 30, and 100 µg/ml) treated macrophages, in a concentration-dependent manner, were able to decrease their pro-inflammatory phenotype when exposed to high glucose and LPS, evident from the significantly decreased activity of NF-κB, TNF-α, and IL-1β (**Figures 5D–F**). Similar findings were observed in the Metformin (2 mM) treated group as well (**Figures 5D–F**). Taken together, these results suggest that Madhugrit has potentials to reduce persistent inflammatory phenotype of macrophages exposed to high glucose and bacterial endotoxins, in chronic diabetic conditions.

### Madhugrit normalized wound healing process under high glucose enviornment

**Figure 6:**
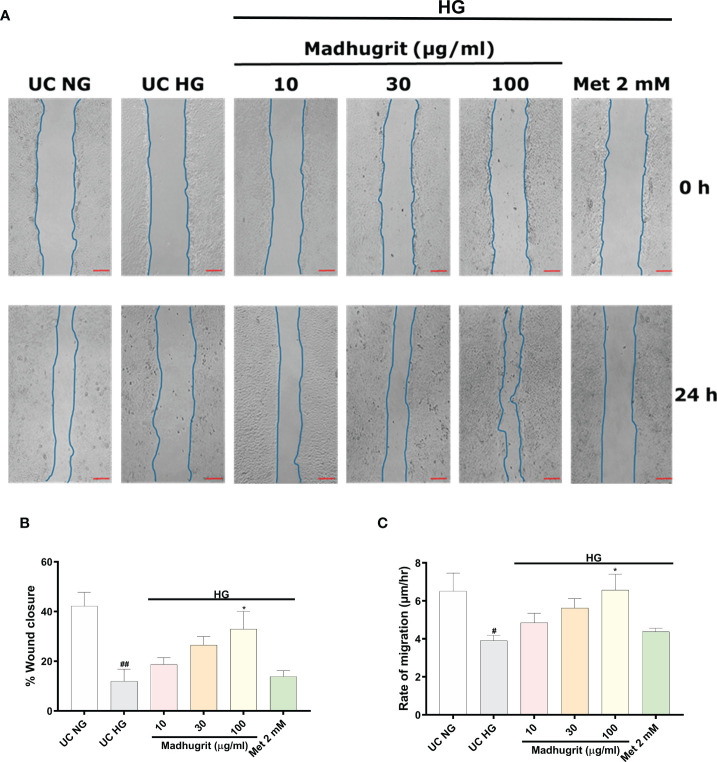
Madhugrit enhanced keratinocyte (HaCaT) migration capacity. **(A)** Representative pictures of scratched wound of HaCaT monolayers (0 and 24 hr) placed in normal glucose (NG) or high glucose (HG) conditions in presence of Madhugrit (10, 30, and 100 µg/ml) or Metformin (2 mM). Blue lines represent wounded area. Scale bar (red) = 100 µm. **(B, C)** Graph quantifying the average wound closure and rate of migration of the wounded HaCaT cells in presence of Madhugrit (10, 30, and 100 µg/ml) or Metformin (2 mM). The statistical significance of the observed differences in the means compared to the UC NG group was analyzed through one-way ANOVA followed by Dunnett’s multiple comparison test and represented as # (p<0.05) or ## (p<0.01). Comparison of the treatments with UC HG group was represented as * (p<0.05).

One of the common complication of diabetes is delayed and impaired wound healing process (46). A controlled inflammation and re-epithelization of the wound are paramount for effective wound healing. Keratinocyte migration, which is an integral part of re-epithelization is inhibited in high glucose conditions (13). Based on Madhugrit’s ability to resolve inflammation, we sought to examine its activity on wound healing under hyperglycemic conditions. As the HaCaT cells were induced with mitomycin C, the cell proliferation process was inhibited to remove any confounding observations related to the rate of migration. The HaCaT cells under high glucose (25 mM) condition displayed a visible defect in the wound healing process. In comparison, the cells treated with Madhugrit (10, 30, and 100 µg/ml) showed a prominent ability for wound healing, but it was not apparent in the Metformin (2 mM) treated group (**Figure 6A**). Based on the image analysis a significant (p <0.05) decrease in the percentage of wound closure and rate of cell migration was observed when HaCaT cells were kept in high glucose conditions (**Figures 6B, C**). Upon Madhugrit treatment, the cells started to display effective wound healing in a concentration-dependent manner. Notably, at 100 µg/ml concentration of Madhugrit the rate of migration and percentage of wound closure was equivalent to that of HaCaT cells kept in normal glucose (5.5 mM) condition (**Figures 6B, C**). These results suggest that Madhugrit has a potential to normalize the wound healing process in a high glucose condition.

### Madhugrit decreased the toxic effects on progeny and aberrant behavior of *C. elegans* in high glucose condition

**Figure 7:**
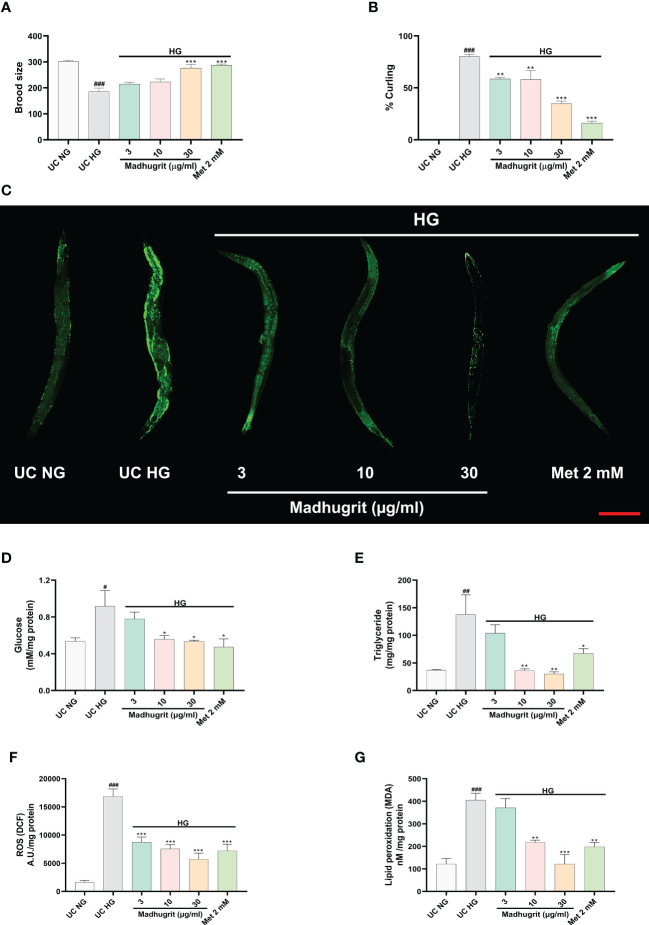
Madhugrit staved off the glucose toxicity markers in *C. elegans* exposed to high glucose (HG) conditions. **(A)** Brood size of the nematodes placed under HG was determined in presence or absence of Madhugrit (3, 10, and 30 µg/ml) or Metformin (2 mM). **(B)** Aberrant behavior of the worms in HG was observed in the form of curling and the number of worms in curled state were quantified in presence or absence of Madhugrit (3, 10, and 30 µg/ml) or Metformin (2 mM). **(C)** Lipid deposition (green) in the nematodes exposed to HG was qualitatively determined by Nile red staining in presence of different treatments. Scale bar (red)=200 µm. **(D-G)** Glucose, triglyceride accumulation, ROS generation and MDA levels in the nematodes were evaluated in presence in presence or absence of Madhugrit (3, 10, and 30 µg/ml) or Metformin (2 mM). The statistical significance of the observed differences in the means compared to the UC NG group was analyzed through one-way ANOVA followed by Dunnett’s multiple comparison test and represented as # (p<0.05), ## (p<0.01), or ### (p<0.001). Comparison of the treatments with UC HG group was represented *, ** or *** depending on whether the calculated p value was <0.05, <0.01 or <0.001.

A constant level of high glucose leads to multi-organ dysfunction which underlies the complications of diabetes (47). To confirm the potency of Madhugrit *in vivo*, the non-mammalian model of *C. elegans* was utilized as the effects of glucose toxicity have been previously reported on the nematode (25). The effect of standalone Madhugrit (3-30 µg/ml) treatment on Brood size was evaluated which showed that it had no toxic effect (data not shown). Further experiments were performed using the same concentrations of Madhugrit. The worms exposed to high glucose (100 mM) showed a significant (p <0.001) reduction in their brood size but the worms exposed to 30 µg/ml concentration of Madhugrit were able to significantly (p <0.001) counteract the negative effects of glucose on their egg laying capacity. Similar effects were observed in the case of Metformin (**Figure 7A**). Under high glucose exposure, *C. elegans* displayed aberrant behavior like curling. Nearly, 80% of the worms were observed to have curling behavior due to glucose-induced stress. Madhugrit (3, 10, and 30 µg/ml) treated worms showed a decrease in the incidences of curling behavior in a concentration-dependent manner (**Figure 7B**). The positive control drug Metformin (2 mM) also reduced % curling in the high glucose exposed worms. The observed increase in brood size and a decrease in % curling suggests that Madhugrit has a strong potential to negate the effects of glucose toxicity on normal physiology.

### Madhugrit normalized the glucose and lipid levels in *C. elegans* under high glucose exposure

The qualitative assessment of the lipid content post glucose exposure and Madhugrit treatment was evaluated by Nile red staining. The worms exposed to 100 mM glucose clearly showed increased lipid accumulation compared to worms grown without added glucose. Madhugrit (3, 10, and 30 µg/ml) treated worms showed a visible reduction in lipid content in a dose-dependent manner. Worms with Metformin (2 mM) treatment also displayed less lipid deposition (**Figure 7C**). Furthermore, the worms exposed to high glucose (100 mM) showed a significant (p <0.05) increase in their glucose (0.92 mM/mg protein) and triglyceride (137.8 mg/mg protein) levels compared to worms grown without added glucose (**Figures 7D, E**). Such biochemical alterations were significantly reduced in the Madhugrit (3, 10, and 30 µg/ml) treated nematodes. Metformin (2 mM) treated worms also efficiently resisted the biochemical changes in response to high glucose exposure. The worms treated with 30 µg/ml of Madhugrit had nearly normalized levels of glucose and triglyceride levels (**Figures 7D, E**).

### Madhugrit decreased ROS levels and lipid peroxidation in *C. elegans* exposed to high glucose

The evaluation of changes in ROS levels was done by the normalized fluorescence intensity of dichlorofluorescein (DCF) (48). High glucose exposure significantly (p <0.001) increased the DCF fluorescence compared to worms grown without added glucose (**Figure 7F**). Madhugrit (3, 10, 30 µg/ml) treated nematodes were able to counteract the development of ROS in response to high glucose as observed by the dose-dependent decrease in fluorescence (**Figure 7F**). An increase in ROS causes the overproduction of MDA, an indicator of oxidative stress (49). The worms exposed to 100 mM glucose displayed a significant (p <0.001) increase in MDA (404.9 nM/mg protein) levels (**Figure 7G**). Whereas, Madhugrit (3, 10, 30 µg/ml) treated worms were able to offset the generation of oxidative stress in a dose-dependent fashion (**Figure 7G**). The 30 µg/ml Madhugrit treated worms had MDA (121.4 nM/mg protein) levels equivalent to the worms grown without added glucose (121.3 nM/mg protein). Collectively, these results implied that Madhugrit possesses robust anti-oxidant properties.

## Discussion

Globally, the pandemic of obesity, inappropriate diet, and sedentary lifestyle has led to an increase in the prevalence of diabetes, decade after decade (50). In 2015 the global economic burden of diabetes was estimated to be more than $1.3 trillion (51). The current pharmacotherapy for diabetes involves insulin secretagogues, insulin sensitizers, alpha-glucosidase inhibitors, biguanides, incretin mimetics, sodium-glucose co-transport-2 inhibitors, amylin antagonists, and insulin. Monotherapy of conventional oral hypoglycemic agents often fail to achieve therapeutic goals and so the use of dual drug therapy is on the rise. Such combinatorial therapies lead to increased incidences of adverse effects like diarrhoea, lactic acidosis, and hepatotoxicity which leads to a decline in patient compliance (52, 53). Anti-diabetic agents from ethnopharmacological origins may be an attractive alternative due to their low cost, better safety profile, and other beneficial pleiotropic effects. Additionally, they might alleviate metabolic abnormalities and abrogate the development of comorbidities associated with diabetes (6).

The present study aimed to characterize the phytochemistry and bioactivity of the Ayurvedic anti-diabetic medicine Madhugrit, a herbo-mineral formulation composed of extracts from 29 herbs and a mineral pitch. The phytometabolite characterization of Madhugrit revealed the presence of several bioactive compounds namely gallic acid, magnoflorine, corilagin, piperine, methyl gallate, rutin, ellagic acid, protocatechuic acid, coumarin, cinnamic acid, and palmatine. These phytochemicals have been reported to possess anti-diabetic, anti-inflammatory, antioxidant, and wound healing properties (6, 7, 54). To assess the collective pharmacological effects of these phytometabolites, we determined the effects of Madhugrit on various *in vitro* models of diabetes and related complications. After establishing the therapeutic properties of Madhugrit we determined its effectiveness on a non-mammalian *in vivo* model of the nematode *C. elegans*. Thus, various *in vitro* and *in vivo* methods were employed to determine the therapeutic potentials of Madhugrit.

Before beginning pharmacological screening of Madhugrit its effect on cell viability was evaluated on rat pancreatic acinar cells (AR42J), rat skeletal myotubes (L6), human monocyte and macrophage (THP-1) cells, and human epidermal keratinocytes (HaCaT). Madhugrit was found to be viable at all physiologically relevant concentrations. The cell viability profile of Madhugrit allowed us to rule out any confounding bias in our obtained results. In order to assess the anti-diabetic properties of Madhugrit, we mainly studied its effect on α-amylase release. Post-prandial hyperglycemia is one of the major symptoms of diabetes. As amylase helps in the breakdown of complex carbohydrates obtained from diet to simple sugars, it promotes the rise of glucose levels in the blood. Therefore, amylase inhibition can help in the effective control of raised glucose levels after meals (10, 32, 33). Madhugrit was found to retard the release of α-amylase from rat pancreatic acinar cells post a starch load. Upon confirmation of the α-amylase inhibitory activity of Madhugrit, we further evaluated its effect on glucose uptake as both work in synergy to maintain euglycemia. The potential of Madhugrit to enhance glucose uptake was performed on rat skeletal myotubes using Metformin as a positive control. It was found that Madhugrit substantially improved the glucose uptake activity of L6 cells as observed by the uptake of fluorescent glucose analog 2-NBDG. The hypoglycemic properties of Madhugrit can be attributed to the presence of gallic acid as one of its major phytoconstituents (55). Gallic acid *in vitro*is reported to directly inhibit α-amylase activity in an *in vitro* model system (56). A study described that when isolated rat skeletal muscles were treated with gallic acid-rich Pu-erh tea extract, glucose transport was stimulated by the enhanced insulin signal transduction in the absence of insulin. This raises the possibility that gallic acid, a major content in Madhugrit, might be an insulin-mimetic agent (57).

Inflammation of the intestinal epithelium observed in obese diabetic patients is reportedly associated with changes in gut microflora. It has been reported that LPS derived from gut microbiota is involved in the onset and progression of the chronic low-grade inflammation observed in obesity (58). Furthermore, the activation of various inflammatory signaling networks initiates the monocyte to macrophage differentiation (28). The present data confirmed that Madhugrit might be able to decrease the LPS-mediated inflammation as observed by the reduced release of TNF-α and IL-6 from the LPS-stimulated THP1 macrophages. Moreover, the conversion of THP1 monocytes to macrophages in the presence of PMA is also reduced by Madhugrit. Such properties of Madhugrit might be able to stave off diabetes-related complications like metabolic endotoxemia and atherosclerosis (27, 28, 58). These pleiotropic effects of Madhugrit can be correlated with the presence of corilagin which is known to be effective in the prevention of LPS-induced inflammation by the inhibition of TLR4 involved in the inflammatory cascade (59, 60).

Inflammation related to diabetes seems to be involved in the development of renal, ophthalmological, cardiovascular, and several other co-morbidities (61). Macrophages under hyperglycemic conditions constantly express a pro-inflammatory phenotype even in the absence of any infection or tissue damage (3). A constant high glucose exposure to macrophages enhances NF-κB activity which further augments TNF-α and IL-1β activity (3, 62–64). In our study, we found that high glucose primed THP1 macrophages even after the LPS challenge, displayed a considerably decreased activity of NF-κB, TNF-α, and IL-1β in presence of Madhugrit. This validates that Madhugrit might possess a potent anti-inflammatory property in addition to being an effective anti-diabetic agent. The presence of magnoflorine and methyl gallate in Madhugrit might explain its potent anti-inflammatory effects. Magnoflorine has been reported to ameliorate inflammation in the *in vivo* diabetic nephropathy model in rats (65). Also, methyl gallate is known to modulate the NF-κB signaling pathway which is majorly responsible for pro-inflammatory cytokine release (66). Thus, in combination, both the phytochemicals might be more effective in lessening the burden of inflammation-related diabetic complications.

Delayed wound healing in diabetes is mainly described as persistent inflammation and an imbalance in extracellular matrix regulation. The production of various growth factors is compromised which results in malfunctioning of cell migration and wound re-epithelization (3, 67). Chronic wounds are highly prone to infection, which can eventually lead to septicemia and other morbidities (68). As Madhugrit considerably staved off inflammation we further investigated its effects on keratinocyte migration, a key process of wound-healing which is also hampered in hyperglycemic conditions (13, 46). When a scratch was applied on the monolayer of high glucose exposed human epidermal keratinocytes (HaCaT cells), a visible reduction of wound closure was observed which was in response to the impaired keratinocyte migration. Madhugrit was able to enhance the migration of keratinocytes exposed to high glucose and accelerate wound closure. This effect of Madhugrit might be due to the presence of a plethora of phytochemicals namely ellagic acid, coumarin, cinnamic acid, and palmatine which are known to enhance wound healing (69–72). Also, it has been reported that cinnamic acid-based formulations in addition to wound healing, have antimicrobial properties as well (73). This further augments the therapeutic potential of Madhugrit.

A prolonged hyperglycemic state leads to the formation of glycated proteins and AGEs which are responsible for various co-morbidities due to the loss of function in proteins in response to crosslink formation. Moreover, AGEs also cause tissue injury by enhancing oxidative stress and inflammation (74). Madhugrit inhibited the formation of AGEs which further bolsters its effectiveness as an anti-diabetic agent. Rutin, a major phytochemical in Madhugrit is known to ameliorate AGEs formation as observed in an *in vitro* model system of protein glycation (75).

After we established the pharmacological effects of Madhugrit using various *in vitro* models we sought to further evaluate its effectiveness in a whole-body organism. Further evaluations were then performed *in vivo* on *C. elegans*. In contrast to *in vivo* rodent models, the short lifespan of the nematode makes it an ideal model to study the detrimental effects of high glucose exposure (76). The insulin signaling pathway is conserved across diverse metazoan. In *C. elegans* the pathway regulates fat storage and reproduction. Disturbances in this pathway are a major contributor to the pathogenesis of obesity and diabetes, and the use of the nematode can be done to study the effects of anti-diabetic agents that may modulate this pathway. Moreover, various natural phytochemicals like quercetin have been reported to prevent high-glucose-induced toxicity in *C. elegans* (77). One of the preliminary physiological defects observed in high glucose exposed *C. elegans* was a decrease in the brood size and aberrant behavior like curling. But these effects were observed in a low frequency in Madhugrit treated groups. Similar attenuation of high glucose-induced reduction in brood size is reported after treatment of worms with leaves of *Aquilaria crassna* (agarwood) (78). Secondly, we observed that high levels of glucose accumulation in the nematode promoted lipogenesis and enhanced triglyceride formation which was visually inspected by Nile red staining. This was not observed in the case of Madhugrit treatment as the worms displayed a normalized lipid deposition. Akin to our findings one study reported that *Apios americana* Medik flower extract treated *C. elegans* showed a drastic reduction in glucose and lipid levels under hyperglycemic conditions (79). A study on the flavonoid-rich extracts of *Citrus aurantium* L. var. *amara* Engl. also showed that the extract-fed *C. elegans* lowered lipid accumulation in the nematodes by affecting the fatty acid synthesis pathway (80). Here, the lipid and glucose-lowering bioactivity of Madhugrit can be correlated with the presence of piperine as one of its constituents (81). It has been documented that high-glucose conditions result in a substantial accumulation of modified mitochondrial proteins and a steady increase in ROS formation in *C. elegans* (82). Hence, we also evaluated the potency of Madhugrit against the formation of ROS and lipid peroxidation products like MDA in high glucose-exposed *C. elegans*. The observed decrease in the ROS and MDA levels can be attributed to the presence of protocatechuic acid as phenolic compounds have been reported to reduce the ROS generation in *C. elegans* (83, 84).

In summary, Madhugrit was found to have robust anti-diabetic, anti-inflammatory, and antioxidant properties which were assessed in various *in vitro* models and on a nematode model. Most of the pharmacological effects of Madhugrit were at par with that of Metformin, the classical anti-diabetic agent (29, 30) but Madhugrit displayed a superior wound healing and anti-oxidant ability than Metformin. Also, due to adverse effects of Metformin, like gastrointestinal disturbances, pancreatitis, hepatitis, vitamin B12 and coagulation abnormalities, and reactive hypoglycemia make its long-term use rather limited (85). Collectively, Madhugrit was able to display a myriad of pharmacological actions against diabetes and related complications. This study also advocates detailed clinical investigations of Madhugrit on human subjects of hyperglycemia and associated co-morbidities.

## Conclusion

This study explored the anti-diabetic, anti-inflammatory, anti-oxidant, wound healing, and lipid-lowering capacity of the ayurvedic prescription medicine Madhugrit. The α-amylase inhibitory and glucose uptake-enhancing properties of the phytomedicine support its purported use as a glucose-lowering agent. The ability of Madhugrit to abrogate pro-inflammatory cytokine production *via* modulation of the NF-κB pathway and hindering the monocyte to macrophage conversion hints towards its use as an effective anti-inflammatory agent as well. The enhanced rate of migration of epidermal keratinocytes under hyperglycemic conditions in presence of Madhugrit evidences its use in the mitigation of diabetic wounds and ulcers. The *in vivo* observations of reduced triglyceride and glucose accumulation, and a decrease of ROS and MDA levels in high glucose-induced *C. elegans* state that Madhugrit might also be able to mitigate the co-morbidities associated with diabetes. Furthermore, given the normalization of brood size and lack of aberrant behavior in the Madhugrit-fed nematodes, it can be acknowledged that the phytomedicine might help retain normal physiological functions under hyperglycemic conditions. Therefore, Madhugrit could be utilized as an effective medicinal product to manage diabetes and associated co-morbidities.

## Data availability statement

The raw data supporting the conclusions of this article will be made available by the authors, without undue reservation.

## Author contributions

AB: Conceptualization, planning, visualization, supervision, writing - review & editing. VG: Conceptualization, planning, visualization, methodology, investigation, data curation, formal analysis, writing original draft. NP: Methodology, investigation, formal analysis. MT: Methodology, investigation, formal analysis. MR: Methodology, investigation, formal analysis. RD: Data curation, writing - review & editing, visualization, project administration, supervision. AV: Writing - review & editing, project administration, conceptualization, visualization, supervision. All authors contributed to the article and approved the submitted version.

## Funding

This research work was funded internally by Patanjali Research Foundation Trust, Haridwar, India.

## Acknowledgments

We extend our gratitute to Dr. Tapan Dey, Ms Moumita Manik, Ms. Deepika Rajput, Mr. Sudeep Verma and Dr. Jyotish Srivastava for their support in the analysis. We are also grateful to Mr. Tarun Rajput, Mr. Gagan Kumar, and Mr. Lalit Mohan for their swift administrative support.

## Conflict of interest

The test article was provided by Divya Pharmacy, Haridwar, Uttarakhand, India. AB is an honorary trustee in Divya Yog Mandir Trust, which governs Divya Pharmacy, Haridwar. In addition, he holds an honorary managerial position in Patanjali Ayurved Ltd., Haridwar, India. Other than providing the test formulation (Madhugrit), Divya Pharmacy was not involved in any aspect of the research reported in this study.

The remaining authors declare that the research was conducted in the absence of any commercial or financial relationships that could be construed as a potential conflict of interest.

## Publisher’s note

All claims expressed in this article are solely those of the authors and do not necessarily represent those of their affiliated organizations, or those of the publisher, the editors and the reviewers. Any product that may be evaluated in this article, or claim that may be made by its manufacturer, is not guaranteed or endorsed by the publisher.

## Abbreviations

UHPLC, Ultra-High Performance Liquid Chromatography; NF-κB, Nuclear factor kappa-light-chain-enhancer of activated B cells; TNF-α, Tumour Necrosis Factor alpha; IL-1β, Interleukin 1 beta; IL-6, Interleukin 6; ROS, Reactive oxygen species; AGEs, Advanced glycation end products; LPS, Lipopolysaccharide; NGM, Nematode growth medium; MDA, Malondialdehyde; TBA, Thiobarbituric acid; TCA, Trichloroacetic acid; Met, Metformin.
