# Supplementary material for: Anti-hyperglycemic contours of Madhugrit are robustly translated in the Caenorhabditis elegans model of lipid accumulation by regulating oxidative stress and inflammatory response
Source: Front Endocrinol (Lausanne). 2022 Dec 5;13:1064532. doi: 10.3389/fendo.2022.1064532 (PMC9762483; doi:10.3389/fendo.2022.1064532)
Supplement: Supplementary file 1 [file Table_1.docx]

Supplementary Material

**Supplementary Table 1**

| **Sr. no.** | **Botanical name** | **Common name** | **Part used** | **Amount (mg)/ tablet** |
| --- | --- | --- | --- | --- |
| 1 | *Accorus calamus* | Sweet flag | Rhizome | 11 |
| 2 | *Cyperus rotundus* | Nut grass | Rhizome | 11 |
| 3 | *Cedrus deodara* | Deodar | Heart wood | 11 |
| 4 | *Curcuma longa* | Turmeric | Rhizome | 11 |
| 5 | *Aconitum heterophyllum* | Indian Ateech | Root | 11 |
| 6 | *Berberis aristata* | Indian barberry | Stem/root | 11 |
| 7 | *Piper longum* | Indian long pepper | Root | 11 |
| 8 | *Plumbago zeylanica* | Wild leadwort | Root bark | 11 |
| 9 | *Operculina turpethum* | Turpeth | Root | 11 |
| 10 | *Baliospermum montanum* | Wild castor | Root | 11 |
| 11 | *Cinnamomum tamala* | Indian bay leaf | Leaf | 11 |
| 12 | *Cinnamomum zeylanicum* | Ceylon cinnamon | Bark | 11 |
| 13 | *Elettaria cardamomum* | Green cardamom | Seed | 11 |
| 14 | *Coriandrum sativum* | Cilantro | Fruit | 11 |
| 15 | *Terminalia chebula* | Black myrobalan | Fruit Rind | 11 |
| 16 | *Terminalia belerica* | Beleric | Fruit Rind | 4 |
| 17 | *Emblica officinalis* | Indian gooseberry | Fruit | 4 |

| **Sr. no.** | **Botanical name** | **Common name** | **Part used** | **Amount (mg)/ tablet** |
| --- | --- | --- | --- | --- |
| 18 | *Piper retrofractum* | Javanese long pepper | Stem | 4 |
| 19 | *Embelica ribes* | Baobarang | Fruit | 4 |
| 20 | *Scindapsus officinalis* | Gajpipul | Fruit | 4 |
| 21 | *Zingiber officinale* | Ginger | Rhizome | 5 |
| 22 | *Piper nigrum* | Black pepper | Fruit | 5 |
| 23 | *Piper longum* | Indian long pepper | Fruit | 5 |
| 24 | *Tinospora cordifolia* | Heart-leaved moonseed | Stem | 100 |
| 25 | *Citrullus colocynthis* | Bitter cucumber | Root | 10 |
| 26 | *Momordica charantia* | Bitter melon | Fruit | 50 |
| 27 | *Swertia chirata* | Felworts | Plant | 50 |
| 28 | *Asparagus racemosus* | Spiny Asparagus | Root | 25 |
| 29 | *Withania somnifera* | Winter cherry | Root | 25 |
| 30 | *Asphaltum punjabianum* | Mineral pitch | Exd. | 50 |

**Supplementary Table 2**

| **Sr.no.** | **Hindi Name** | **Name** | **Book Ref.** | **Page No.** | **Amount (mg/tablet)** |
| --- | --- | --- | --- | --- | --- |
| 1 | Chandraprabha Vati | Classical Medicine | B.R | 730 | 200 |
| 2 | Giloy Dry Extract | *Tinospora cordifolia* | B.P.N | 270 | 100 |
| 3 | Indrayana Dry Extract | *Citrullus colocynthis* | B.P.N | 389-390 | 10 |
| 4 | Karela Dry Extract | *Momordica charantia* | B.P.N | 584 | 50 |
| 5 | Chirayata Dry Extract | *Swertia chirata* | B.P.N | 73 | 50 |
| 6 | Shatavar Dry Extract | *Asparagus racemosus* | B.P.N | 392-393 | 25 |
| 7 | Ashwagandha Dry Extract | *Withania somnifera* | B.P.N | 393-394 | 25 |
| 8 | Shuddh Shilajit | *Asphaltum punjabianum* | B.P.N | 612 | 50 |

**B.R:** Bhaisajya Ratnavali; **B.P.N:** Bhavprakash Nighantu
